# Relief of vasospasm with fasudil after off-pump coronary artery bypass grafting: a case study

**DOI:** 10.1186/s40792-018-0481-9

**Published:** 2018-07-27

**Authors:** Akira Fujita, Hiroshi Kurazumi, Ryo Suzuki, Masaya Takahashi, Akihito Mikamo, Kimikazu Hamano

**Affiliations:** Department of Surgery and Clinical Science, Graduate School of Medicine, Yamaguchi University, 1-1-1 Minami-Kogushi, Ube, Yamaguchi 755-8505 Japan

**Keywords:** Coronary artery vasospasm, Coronary artery bypass graft, Rho kinase inhibitor, Fasudil

## Abstract

**Background:**

Coronary vasospasm after coronary artery bypass grafting (CABG) is a rare but potentially lethal complication. It is often refractory to several vasodilators. We report a case of refractory coronary vasospasm relieved by fasudil injection.

**Case presentation:**

A 74-year-old woman who had three instances of in-stent stenosis at the left anterior descending artery (LAD) was referred for CABG treatment. Preoperative coronary angiography showed 90% in-stent stenosis of the proximal LAD and 75% stenosis of the diagonal branch. We performed a left internal thoracic artery (LITA)-LAD bypass and a right internal thoracic artery (RITA) diagonal branch bypass. After anastomosis, transit time flow measurement revealed poor blood flow of LITA-LAD bypass even after re-anastomosis. We performed coronary angiography and detected a vasospasm in the native coronary arteries, which was not relieved using conventional vasodilators (calcium channel blockers, isosorbide dinitrate, and nicorandil) However, we were able to relieve the coronary vasospasm by administering fasudil (a Rho kinase inhibitor) injection without causing systemic hypotension.

**Conclusions:**

Fasudil may be an important vasodilator, especially in cases of coronary vasospasm after CABG.

## Background

Coronary vasospasm is a rare but serious complication of coronary artery bypass grafting (CABG). Coronary vasospasm is generally treated using calcium channel blockers, such as isosorbide dinitrate and nicorandil, but is often refractory to these vasodilators. We report a case of refractory coronary vasospasm that was relieved using selective injections of fasudil.

## Case presentation

**Fig. 1:**
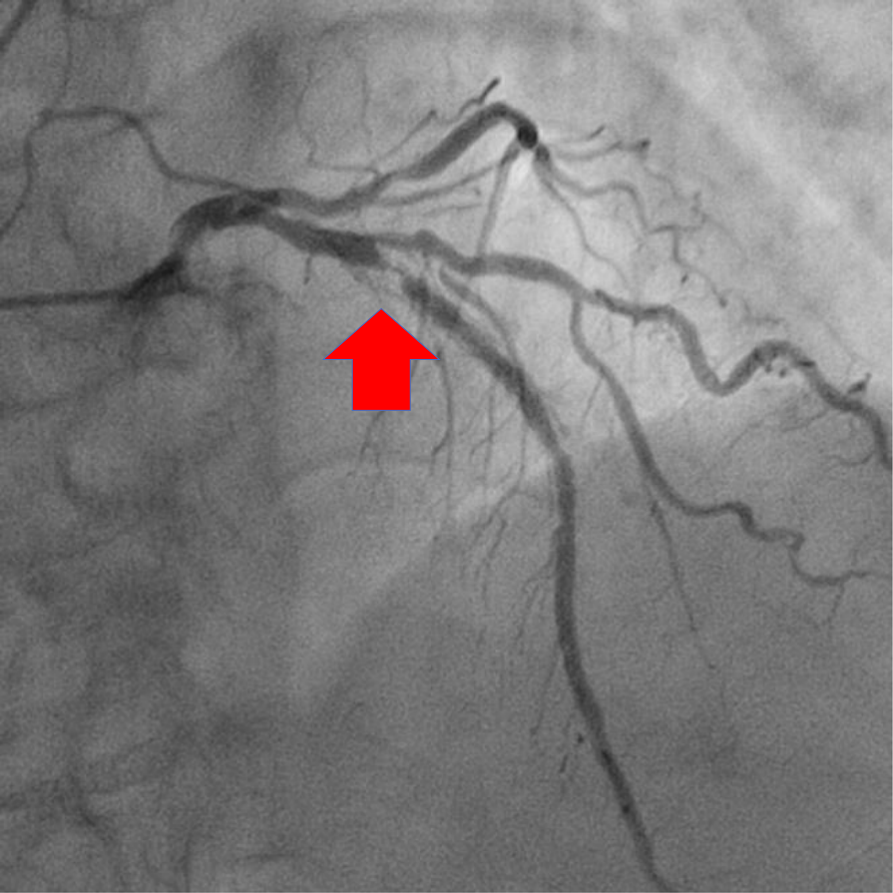
Preoperative coronary angiography revealed 90% in-stent stenosis of the proximal left anterior descending artery (arrow) and 75% stenosis of the diagonal branch

A 74-year-old woman was referred for CABG treatment. She had a history of diabetes mellitus and dyslipidemia and previously underwent percutaneous stenting of the mid right coronary artery and the proximal left anterior descending artery (LAD). Preoperative coronary angiography revealed 90% in-stent stenosis of the proximal LAD and 75% stenosis of the diagonal branch (Fig. 1). In addition, she had three instances of in-stent stenosis at the LAD. Whenever restenosis was diagnosed, the implementation of percutaneous coronary intervention (PCI) was repeated. Taking this history into consideration, we decided to perform a left ITA (LITA)-LAD bypass and a right ITA (RITA) diagonal branch bypass. The ITAs were mobilized as skeletonized grafts. We routinely used nicorandil (4 mg/h) and diltiazem (4 mg/h) during CABG operation for the prevention of vasospasm.

At first, we performed RITA diagonal bypass. Subsequently, we performed anastomosis of LITA-LAD bypass. After CABG, the patient had stable circulation (BP 126/54 mmHg, HR 62 bpm) without changes in ST segment as monitored by electrocardiogram. When we examined blood flow of the RITA diagonal bypass, transit time flow measurement revealed reasonable blood flow (flow rate 20 mL/min, pulsative index 3.4, diastolic flow of 82%). On the other hand, the LITA graft showed comparatively poorer blood flow (flow rate 15 mL/min, pulsative index 2.1, diastolic flow 74%) than the RITA graft. Flow competition between the RITA and LITA was unlikely to occur considering the location of the stenotic lesion. In addition, taking into the consideration the perfused region of the LAD and the severe stenosis in the stent, the graft blood flow was too low and technical anastomotic stenosis was suspected. We re-anastomosed the LITA-LAD bypass. However, even after re-anastomosis of LITA-LAD bypass, transit time flow measurement revealed worsening of graft flow compared to before (LITA-LAD: flow rate 7 mL/min, pulsatile index 4.8, diastolic flow 68%; RITA diagonal: flow rate 11 mL/min, pulsatile index 5.6, diastolic flow 76%). We immediately closed the wound and moved the patient to a hybrid operating room to examine the causes of this progressively low graft flow.

**Fig. 2:**
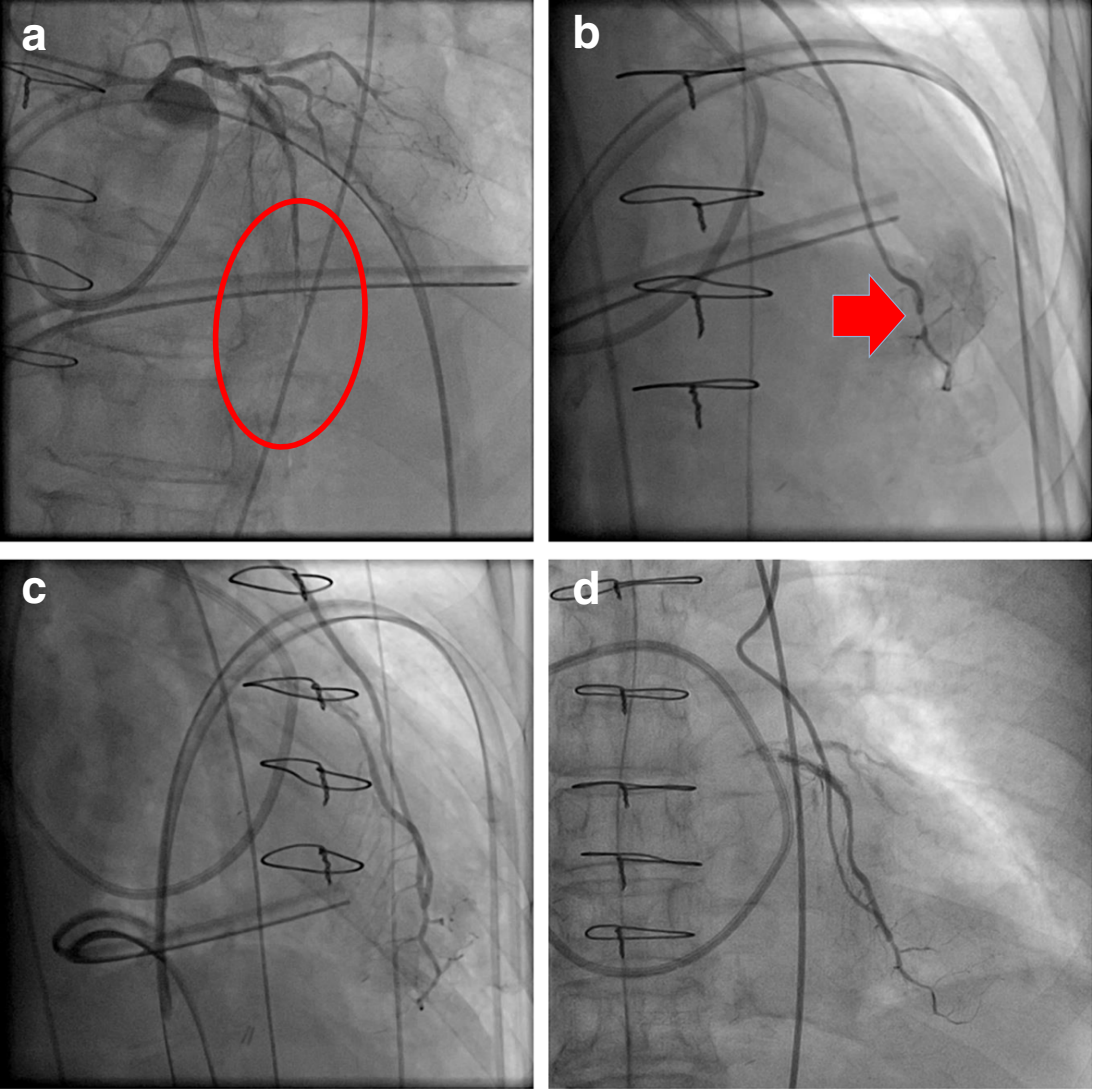
Coronary angiography performed in a hybrid operation room. **a**, **b** Vasospasm in the left anterior descending artery (circle and arrow). **c** The fasudil injection was successful and **d** coronary angiography revealed a patent bypass graft on postoperative day 1

We performed coronary angiography and detected vasospasms in the native coronary arteries (Fig. 2a, b) without ST elevation, as seen on the electrocardiogram. We performed intracoronary injections of verapamil (5 mg) and isosorbide dinitrate (6 mg) through the ITA graft, but no improvement was observed. We subsequently injected fasudil (20 mg) through the LITA and observed that coronary flow through the LITA graft improved (thrombolysis in myocardial infarction risk score of 3) (Fig. 2c). The intracoronary fasudil injection did not cause systemic hypotension, as demonstrated by the postinjection measurement of 86/56 mmHg compared with the preinjection measurement of 93/52 mmHg. We subsequently applied intra-aortic balloon pumping to secure coronary blood flow.

On postoperative day 1, coronary angiography revealed a patent bypass graft and sufficient coronary runoff (Fig. 2d). Electrocardiogram showed no ischemic changes. Peak postoperative CK, CK-MB, and troponin T were 582 U/L (41–153 U/L), 7.6 IU/L (4–18 IU/L), and 0.307 ng/mL (0–0.014 ng/mL), respectively. The patient experienced an uneventful clinical course without vasospasm recurrence and was discharged on postoperative day 11.

## Conclusions

Coronary vasospasm is a well-known complication of CABG, with an incidence of approximately 0.8–1.3% [1]. There are several causes of coronary vasospasms, including diabetes mellitus, preoperative use of beta blockers and calcium blockers, perioperative alpha-adrenergic stimulation, vascular manipulation during surgery, hypothermia, hyperventilation, and stimulation using a chest tube [2]. Our patient’s risk factors were diabetes mellitus and the preoperative use of a beta blocker, although these risk factors are common among patients who are undergoing CABG. Furthermore, it can be very difficult to predict the onset of coronary vasospasm based on the patient’s history. Although coronary vasospasm is typically associated with catastrophic circulatory collapse, our patient had relatively stable circulation, and the only diagnostic clue was the low flow in the bypass graft. Stable circulation might be affected by ischemic preconditioning caused by severe stenosis of the proximal LAD.

It is recommended that a bypassed ITA graft should be 20 mL/min or more. In our case, LITA-LAD bypass initially showed a blood flow of 15 mL/min. That is a marginal level for predicting ITA graft failure using transit time flow measurement [3]. Additionally, it was reported that a bypassed graft had more blood flow when anastomosed at a distal site with more severe stenotic lesion [3]. Thus, we were able to consider that decreased LITA-LAD flow compared to that of the RITA diagonal branch was abnormal. Low graft blood flow is one of the predictive factors for graft failure. It should be treated immediately. We were able to move to a hybrid operating room because the patient’s circulation was stable. If CABG had been performed in a hybrid operating room, the diagnosis of coronary vasospasm would have been sooner.

Calcium sensitivity relates to the onset of coronary vasospasm. In this context, Rho kinase plays an important role, which has been determined in animal studies [4]. In the present case, the intraoperative serum calcium level was within the normal range, which did not change before and after the spasm (preoperation 1.23 mmol/L, intraoperation 1.14–1.19 mmol/L, after-vasodilator injection 1.13 mmol/L, postoperative day 1 1.10 mmol/l). Conventional calcium-dependent vasodilators did not resolve the coronary vasospasm, although fasudil treatment provided dramatic relief. Furthermore, the administration of fasudil injections relieved the LAD vasospasm without causing systemic hypotension.

Recent reports have indicated that fasudil has therapeutic effects in cases of coronary vasospasm [5] and increases blood flow in muscular arterial grafts, such as in the radial and gastroepiploic arteries [6]. Thus, fasudil may be an important vasodilator, especially in cases of coronary vasospasm after CABG. Although IABP and other mechanical supports are also considered for the treatment of coronary vasospasm, particularly in catastrophic circulatory collapse, a Rho kinase inhibitor injection was effective for refractory vasospasm after CABG in our case. Thus, fasudil may be an important vasodilator, especially in cases of coronary vasospasm after CABG.

## Abbreviations

CABG: coronary artery bypass grafting
CK: Creatine kinase
CK-MB: Creatine kinase-muscle/brain
ITA: Internal thoracic artery
LAD: Left anterior descending artery
LITA: Left internal thoracic artery
PCI: Percutaneous coronary intervention
RITA: Right internal thoracic artery
